# By their words ye shall know them: Evidence of genetic selection against general intelligence and concurrent environmental enrichment in vocabulary usage since the mid 19th century

**DOI:** 10.3389/fpsyg.2015.00361

**Published:** 2015-04-21

**Authors:** Michael A. Woodley of Menie, Heitor B. F. Fernandes, Aurelio José Figueredo, Gerhard Meisenberg

**Affiliations:** ^1^Department of Psychology, Technische Universität Chemnitz, ChemnitzGermany; ^2^Center Leo Apostel for Interdisciplinary Studies, Vrije Universiteit Brussel, BrusselsBelgium; ^3^Departments of Psychology and Genetics, Federal University of Rio Grande do Sul, Porto AlegreBrazil; ^4^Department of Psychology, University of Arizona, Tucson, AZUSA; ^5^Department of Biochemistry, Ross University School of Medicine, PortsmouthDominica

**Keywords:** co-occurrence model, intelligence, Flynn effect, WORDSUM, vocabulary

## Abstract

It has been theorized that declines in general intelligence (*g*) due to genetic selection stemming from the inverse association between completed fertility and IQ and the Flynn effect co-occur, with the effects of the latter being concentrated on less heritable non-*g* sources of intelligence variance. Evidence for this comes from the observation that 19th century populations were more intellectually productive, and also exhibited faster simple reaction times than modern ones, suggesting greater information-processing ability and therefore higher *g*. This co-occurrence model is tested via examination of historical changes in the utilization frequencies of words from the highly *g*-loaded WORDSUM test across 5.9 million texts spanning the period 1850–2005. Consistent with predictions, words with higher difficulties (δ parameters from Item Response Theory) and stronger negative correlations between pass rates and completed fertility declined in use over time whereas less difficult and less strongly selected words, increased in use over time, consistent with a Flynn effect stemming in part from the vocabulary enriching effects of increases in population literacy. These findings persisted when explicitly controlled for word age, changing literacy rates and temporal autocorrelation. These trends constitute compelling evidence for the co-occurrence model.

## Introduction

Ever since Galton (1869) forecast declining intelligence on the basis of the shifting demographics of the Victorian population, there has been controversy about the future of human intelligence.

Early use of IQ testing seemed to confirm Galton’s (1869) predictions, as most studies found that IQ was inversely related to fertility, suggesting directional genetic selection for lower intelligence (Lynn, 2011) – a trend that persists into the present (Lynn and van Court, 2004; Meisenberg, 2010; Reeve et al., 2013; Kanazawa, 2014).

In the West, up until the early to mid 19th century, those with high levels of socioeconomic status, wealth, and education (all of which are proxies for intelligence; Herrnstein and Murray, 1994) had higher numbers of surviving offspring relative to those with comparatively lower levels (Clark, 2007; Skirbekk, 2008), suggesting that higher intelligence may have conferred fitness advantages on individuals having to cope with extremes of cold, disease outbreaks, and conflict (Woodley and Figueredo, 2013). Subsequent increases in global temperature, coinciding with the end of the Little Ice Age in the mid-19th century, reduced environmental harshness, boosting agricultural yields thus reducing ecological stress and conflict (see: Zhang et al., 2007, 2011 for a demonstration of the inverse historical relationship between temperature and conflict). This would have substantially relaxed selection against those with lower intelligence (Woodley and Figueredo, 2013). This was coupled with advances in medicine (which would have included better means of fertility control, hygiene, nutrition, and medication; Lynn, 2011), and also social innovations such as welfare, mass schooling, and universal healthcare. The combined effect of these was a demographic transition characterized by general reductions in fertility, which were most pronounced among those with higher intelligence (Lynn, 2011). This was mediated primarily by fertility control coupled with the increasing prevalence of opportunities to delay fertility (i.e., higher education, increasing status competition, etc., which disproportionately attenuated the fertility of high-IQ women relative to men; Low et al., 2002; Meisenberg, 2010).

Child mortality was historically concentrated among those with low socioeconomic status (Geary, 2000) and impacted 50% of all children born in some European regions during the Renaissance – dropping to around 1% in the modern era, starting in the 19th century (Volk and Atkinson, 2008). Reductions in child mortality therefore further boosted the reproductive success of those with low IQ relative to those with high IQ. Additionally, historically high levels of child mortality would also have functioned as a source of purifying selection against *de novo* Single Nucleotide Polymorphisms (SNPs) and other deleterious mutations, which based on present rates of accumulation (i.e., 70 *de novo* SNPs per diploid genome per generation; Kong et al., 2012) should be associated with reproductive failure rates of a similar predicted magnitude to those that were actually observed historically in the child mortality data (i.e., 88%; Keightley, 2012).

The presence of directional selection favoring lower IQ coupled with increasing levels of mutation accumulation stemming from the breakdown of purifying selection should have reduced population-level IQ in the West since the 19th century. Consistent with this expectation, early intelligence researchers predicted that time-series studies of populations would reveal substantial generational declines in intelligence (Lentz, 1927; Cattell, 1937). Despite these predictions, the first such studies revealed that IQ scores had in fact *risen* across time (i.e., Cattell, 1950). This apparent contradiction was even termed “Cattell’s Paradox” (Higgins et al., 1962) after psychometrician Raymond B. Cattell, who in the 1930s was prominent in predicting declining intelligence due to the lower fertility of those with higher intelligence (i.e., Cattell, 1937).

Debate as to the reality of massive secular gains in IQ scores was put to rest in the 1980s by Flynn (1984, 1987), who documented a steady rise in IQ across countries and across IQ batteries averaging three points per decade. The preponderance of the data collected subsequently has reinforced Flynn’s finding (Trahan et al., 2014; Pietschnig and Voracek, 2015). Few now dispute the reality of this ‘Flynn effect’ (Herrnstein and Murray, 1994), however, there remains much debate as to its causes (Williams, 2013; Pietschnig and Voracek, 2015).

In the 1990s Lynn (2011) proposed a solution to Cattell’s Paradox based on the idea that while “genotypic intelligence” (i.e., the theoretical level of intelligence resulting from the action of genes alone) has been declining due to genetic selection, these declines have been massively offset by gains in “phenotypic intelligence” (i.e., the intelligence that results from the interaction between a population’s genes and improved environments). Loehlin (1997) illustrated this with the analogy of rising tides (representing the phenotypic-IQ-boosting effects of improving environments) lifting leaky boats (representing the much smaller losses expected on the basis of selection for lower IQ).

### Anomalies Lead to New Findings

Lynn’s model fails to account for certain observations. For example, if intelligence has been rising overall, why were 19th century Western populations much more innovative on a per capita basis across a wide range of fields (science, technology, mathematics, literature, and philosophy) and also much more generative of geniuses than modern populations (Murray, 2003; Huebner, 2005; Simonton, 2013), despite there being more individuals alive today with greater access to education, hygiene, high-quality nutrition, and other proposed elicitors of the Flynn effect (Williams, 2013)? This may seem like a counter-intuitive proposition, as there are many examples of substantial scientific progress in the modern era (i.e., in fields such as computing, genetics, materials science etc). Nonetheless, while breakthroughs are still occurring, they are occurring at a lower rate than was the case in the past, as are the geniuses responsible for them.

These indices of innovation and genius demonstrating per capita declines are based on the historiometric method of collating notable developments and individuals across many different encyclopedic reference works, first proposed by Galton (1869). There is a striking degree of agreement among different reference works as to precisely what counts as a major innovation, and who was responsible for it, which suggests that this is a robust method for estimating secular trends (Murray, 2003).

Simulated historical trends in “genotypic intelligence” (the population level intelligence change that would be expected due to selection alone, absent the Flynn effect) predict changing rates of innovation and genius (Woodley, 2012; Woodley and Figueredo, 2013). Clearly, there are factors other than intelligence influencing the innovativeness and creativity of populations, such as the presence or absence of key cultural factors and ‘low-hanging fruit,’ that is, novel innovations and discoveries that are easy to make (e.g., Horgan, 1997; Cowen, 2011), however, the findings of Woodley (2012) and Woodley and Figueredo (2013) suggest that losses in intelligence occurring *despite* the Flynn effect may nonetheless have been an important contributing factor to these trends also.

### The Co-occurrence Model: Decreases in *g* and the Flynn Effect Occur Simultaneously

The general intelligence factor, or *g* factor (Spearman, 1904), represents the component of intelligence which all tests of mental ability collectively share – it measures the ability to cope with cognitive complexity and is thus extremely important, as it is better at predicting individual differences in various life outcomes than are the relatively narrower cognitive abilities (Jensen, 1998).

The method of correlated vectors (MCV) compares the *g* loading (i.e., the correlation between a particular ability measure and the *g* factor) of different subtests of an IQ battery with the magnitudes of a set of associated effect sizes by correlating the vector of one with the vector of the other. This indicates the extent to which a given correlate of IQ is associated with *g* or narrower cognitive abilities (Jensen, 1998). When using MCV to examine whether the magnitude of genetic selection against IQ is related to the *g* loadings of subtests, positive correlations have been found (Woodley and Meisenberg, 2013a; Peach et al., 2014). When the same analysis is attempted for the Flynn effect, the effect size is *negatively* related to subtest *g* loadings (te Nijenhuis and van der Flier, 2013). The vectors of genetic and biological factors such as subtest heritabilities, inbreeding depression, reaction times, and indicators of mutation load typically correlate positively with *g* loadings in MCV (Prokosch et al., 2005; Rushton and Jensen, 2010; te Nijenhuis et al., 2014b), whereas the vectors of environmental effects, such as intelligence gains among adopted children, gains via educational interventions and gains via retesting typically correlate negatively with *g* loadings (te Nijenhuis et al., 2007, 2014a, 2015).

This suggests that the *highly heritable g* factor has been declining historically due to genetic selection and accumulating mutations (thus accounting for the apparent high intellectual productivity of 19th century populations relative to modern ones) whereas more *trainable* and *less heritable* specialized abilities exhibiting lower *g* loadings have been increasing in populations over time in response to educational and environmental improvements. Thus, Flynn effects and declines in *g* due to genetic selection and mutation accumulation *co-occur*, albeit *hierarchically* in that selection and mutation affect the top of the latent cognitive ability hierarchy [i.e., Carroll’s (1993) Stratum III] whereas the Flynn effect is restricted to the narrower abilities and test specificities at the bottom of the hierarchy [i.e., Carroll’s (1993) Stratum I; Woodley and Figueredo, 2013].

Evidence for this model has recently been found in data indicating that performance on tests of simple reaction time has been declining since the late 19th century (Silverman, 2010; Woodley et al., 2014a). Simple reaction time, as a measure related to information processing speed, is a culturally neutral biological marker of *g* (Jensen, 2006, 2011). Changing population averages may thus reflect the effects of genetic selection and mutation accumulation on *g*. The change in mean reaction time when scaled in terms of *g*-equivalents, suggests a decline of -1.21 points per decade in the US and UK when the study-means are controlled for various sources of between-study methods variance (Woodley et al., 2014a). This observed loss is similar to the predicted loss derived from combining the results of a meta-analysis of 10 estimates of *g* decline computed on the basis of negative IQ × fertility correlations (-0.39 points per decade) with the additional decline derived from a study examining the effects of paternal age on offspring *g* as a proxy for the generational effects of *de novo* SNPs on *g* (-0.84 points per decade; combined loss = -1.23 points per decade; Woodley of Menie, 2015).

The co-occurrence model has also been tested using cohorts from the Netherlands born between 1950 and 1990. Using MCV on a sample of 63 subtests, it was found that the decline magnitudes among subtests showing *anti-* or “reverse” Flynn effects (i.e., IQ losses presumably due to selection and mutation) were borderline significantly and positively related to their *g* loadings, whereas those showing Flynn effects were not. The average *g* loading of the subtests exhibiting anti-Flynn effects was furthermore higher than that of the subtests exhibiting Flynn effects, consistent with predictions from the co-occurrence model (Woodley and Meisenberg, 2013b).

### A New Test of the Co-occurrence Model

Here a novel test of the co-occurrence model is presented involving an examination of the temporal prevalence of vocabulary words across English-language texts over the past century and a half. Scores on tests of vocabulary, although themselves assessing a specific ability, are typically among the most *g*-loaded measures of IQ and are also among the most heritable (Kan et al., 2013). Nevertheless there is considerable heterogeneity in the difficulties of the vocabulary items that comprise these scales (Beaujean and Sheng, 2010), thus there is scope for testing predictions derived from the co-occurrence model using vocabulary measures at the item level.

An excellent vocabulary measure is WORDSUM (developed by Thorndike, 1942) from the General Social Survey (GSS), which has been administered to ‘household’ samples of the American public on a regular basis since 1974. WORDSUM involves showing the respondent a card containing 10 target words. They must find the synonymous term or phrase among five alternatives.

Wolfle (1980) found that WORDUM performance correlated at 0.71 with full-scale IQ. When this correlation is corrected for the reliability of WORDSUM (0.73; Hout and Hastings, 2012), and psychometric validity (0.90; Jensen, 1998), it rises to 0.93, indicating a very high *g* loading, as is typical of vocabulary measures (Kan et al., 2013).

Attempts at determining whether there is any kind of a secular trend toward changing overall performance on this measure in the GSS have yielded inconsistent results (Haung and Hauser, 1998; Beaujean and Sheng, 2010; Flynn, 2012). Studies by Bowles et al. (2005) and Cor et al. (2012) found that WORDSUM words could be grouped into two classes based on difficulty. Both groups of researchers also found that earlier-born cohorts exhibited higher difficult-vocabulary knowledge relative to more recently-born ones, suggesting declining performance with respect to difficult words. This is consistent with the co-occurrence model, as it is performance on the most difficult (and therefore most *g*-loaded) words that is declining. These trends would furthermore be congruent with the presence of persistent negative associations between IQ and fertility on WORDSUM vocabulary knowledge, which have been found in GSS birth cohorts dating back to 1880–1899 (van Court and Bean, 1985; Lynn and van Court, 2004).

Studies involving large lexical databases, such as Haung and Hauser (1998), who attempted to determine changes in the frequencies of WORDSUM target words across a 1 million word database, and Roivainen (2014), who used Google Ngram Viewer (Michel et al., 2011) to track the frequencies of WORDSUM, WAIS, WAIS-R, WISC, and WISC-R vocabulary words, have also found evidence for declining usage frequencies, especially among more difficult words. Here, the degree to which WORDSUM item-level difficulties, the negative correlation between item pass rates and fertility (a measure of the strength of genetic selection associated with each item), and changing levels of population literacy predict changes in word prevalence across texts from the mid 19th century to the present, is investigated, while controlling for various factors. Based on the co-occurence model, it is predicted that more difficult words should be declining in usage over time and that this decline should in part be predicted by genetic selection. Conversely, there should be a Flynn effect on easier words owing in part to the effects of increasing population level literacy enriching people’s vocabularies coupled with increasing demand for literature containing less cognitively demanding words.

In the present study Google Ngram Viewer is employed in tracking WORDSUM word frequencies. The Ngram viewer provides a database of more than 5 million texts (newspapers, works of fiction, non-fiction, technical works, etc.), comprising more than 500 billion words that can be searched using the target WORDSUM words, thus revealing their year-on-year frequencies. The database has considerable reach in time also – spanning from 1500 to nearly the present.

One major advantage of examining the prevalence of WORDSUM words across texts is that it can be reasonably assumed that the authors of the texts were using these words correctly – hence appearance in print is tantamount to the authors effectively ‘passing’ that item in WORDSUM. This is potentially important as scores on psychometric tests with multiple-choice-type answer formats are known to be inflated by factors relating to test wiseness such as guessing (Brand, 1987; Must and Must, 2013). It has been found that people are more likely to utilize guessing on more *g-*loaded measures of ability (Woodley et al., 2014b). Secular gains due purely to increased guessing therefore potentially weaken the capacity for psychometric tests to directly detect declines on *g* due to genetic selection and mutation accumulation as the effects are occurring on the same variance component (Woodley et al., 2014b). Tracking word usage trends across a representative corpus of written texts therefore yields potentially more *ecologically-valid* data on secular trends, as guessing and other factors associated with the ‘artificiality’ of the testing environment cannot be influencing these trends.

## Materials and Methods

Google Ngram Viewer (Michel et al., 2011) generates estimates of the year-on-year prevalence of the 10 WORDSUM target words across a very large sample of English-language texts scanned by Google. All years between 1850 and 2005 were considered. 1850 was used as the lower cutoff year because in the majority of Western countries fertility began to negatively relate to IQ proxies such as socioeconomic status and education by the middle of the 19th century (Skirbekk, 2008). Ngram Viewer’s algorithm normalizes the frequency of words by the number of books published in each year; therefore the yearly word frequencies are not biased due to increasing amounts of text. All searches were case insensitive (permitting both upper and lower case variants of the same word to be included in the search), and were restricted to the lexical category, grammatical conjugation, and grammatical number specified in WORDSUM, thus Ngram was only searched for the specific WORDSUM target word, rather than variants.

Item difficulties (δ) for the 10 target words were obtained from Beaujean and Sheng (2010), calculated using item response theory (IRT) on individual-level GSS-respondent data totaling 25,555 participants. This is a measure of how difficult each word was to “pass” on the test, and roughly reflects the relative proportion of the calibration sample that got each word correct.

The correlations between fertility and item-level pass rates (henceforth *r*FPR) were also computed for each word among the white subset of the GSS that had completed fertility for males and females separately and then averaged (i.e., the subset aged 41 and older; *N* = 4,252 for females, *N* = 3,542 for males, total *N*= 7,794). Using the white subsample ensured that the fertility patterns among the demographic that historically contributed disproportionately to literature were captured (Murray, 2003). Each *r*FPR value was disattenuated for unreliability by dividing it by the square-root of the item-level reliabilities reported in Hout and Hastings (2012, Figure 2C, p. 12; these were obtained visually from the figure). All of these coefficients are *negative* in direction indicating that *r*FPR was inversely related to number of children. Furthermore, MCV demonstrates that *r*FPR is *inversely proportional* to the difficulty of each word (*r* = -0.71, *p*< 0.05, *N* = 10), indicating that the *magnitude* of item-level *r*FPR is *positively* related to item difficulty and that genetic selection is strongest on more *g*-loaded measures, replicating previous findings (Woodley and Meisenberg, 2013a; Peach et al., 2014).

A potentially confounding factor influencing word frequencies is word age. Curzan (2009) has argued that older words are likely to be better known to people who read as changes in written language lag behind changes in spoken language. An implication of this is that older words will therefore have higher usage frequencies than younger words simply because they are more established in text, which could influence year-on-year usage frequencies independently of cognitive or other changes. Thus word age is a potentially important factor to control. This was operationalized using data from Merriam-Webster (2010), the *Random House Unabridged Dictionary* (Flexner, 2003), and the *Online Etymology Dictionary* (Harper, 2010) on the year in which the word first appeared in its modern-usage context in the English language (see **Table 1**). Words for which only the decade of first use was available instead of the precise year were assigned the median year of the respective decade.

**Table 1:** WORDSUM item data used in the analyses.

WORDSUM (Word code)	Item difficulty(δ)	Item fertility pass rate correlation (*r*FPR)*	Item-level reliability	Word debut (Year of first use in the Anglo phone literature)
A	-1.17	-0.039	0.60	1275
B	-1.27	-0.038	1.00†	1726
C	1.26	-0.062	0.90	1742
D	-1.13	-0.022	0.75	1602
E	-0.67	-0.082	0.97	1492
F	-0.83	-0.052	0.57	1387
G	0.74	-0.058	0.63	1287
H	0.82	-0.079	0.88	1666
I	-0.92	-0.031	0.74	1437
J	1.04	-0.100	0.76	1546

*All *r*FPR correlations are statistically significant at the *p* ≤ 0.05 level for an *N* of 7,794. †Hout and Hastings (2012) assign an item-level reliability of >1 to word B. This may have resulted from model overfitting therefore the word is assigned a perfect reliability (necessitating no correction) instead. In order to safeguard future General Social Survey (GSS) waves utilizing WORDSUM, the target words are kept confidential. They are therefore represented here with letter codes corresponding to the order in which the items are administered.

Another important contributing factor is literacy. As more people become literate, literature moves out of the domain of a literate elite, into a mass-market dominated by newly literate people of more modest intelligence. Therefore increasing literacy might drive down the demand for cognitively demanding words in addition to enriching people’s vocabularies with respect to less difficult words (consistent with this expectation Must et al. (2003) found that secular literacy gains are more pronounced on less cognitively demanding measures of literacy). Changing literacy was operationalized using a year-on-year average measure of both male and female written literacy rates sourced from the British Library (2001). These data cover the period from 1850 to 1900, when literacy rates reach approximately 99% for both sexes, and apply to the UK, however, historical literacy trends are broadly paralleled across Western countries (Vanhanen, 2009). Written literacy likely also overestimates the proportion of illiterates, as more people historically attained reading literacy than written literacy (British Library, 2001), therefore it constitutes a conservative measure. Given that near maximal literacy (99%) was attained by 1900, this value was assigned to all subsequent years.

As serial measurements with the same experimental subject or unit of analysis (such as the time series of word frequencies) are likely to be auto-correlated, analyses with repeated measurements must account for this. The method of Multi-Level Modeling (MLM) was employed.

The Restricted Maximum Likelihood (REML) procedure was used to estimate the variance and covariance parameters, as this is recommended for fitting mixed models due to nuisance parameters (such as negative estimates of variances that are very close to zero) having no effect upon the estimation (Corbeil and Searle, 1976). The significance tests were conducted with Hierarchical Type I Sum of Squares so that the effect of each predictor was residualized against the effects of prior predictors, according to the theoretically specified order (see **Table 2**). This permitted the effects of time with word difficulty and with *r*FPR upon word utilization frequency to be tested after accounting for their component main effects, as well as after statistically controlling for word age, population literacy and their interactions with other predictors, in addition to within-subject temporal autocorrelations.

**Table 2:** Hierarchical Type I Sum of Squares tests of fixed effects^a^ upon word usage frequencies in the literature.

Predictor	Semipartial *r*
Word debut year	-0.481*
Literacy	0.020*
Ln(Time)	0.049*
Item difficulty(δ)	-0.186*
*r*FPR	0.006*
Literacy × Item difficulty(δ)	-0.062*
*r*FPR × Item difficulty(δ)	-0.105*
Ln(Time) × Item difficulty(δ)	-0.066*
Ln(Time) ×*r*FPR	0.010*
Ln(Time) ×*r*FPR × Item difficulty(δ)	0.032*

δ, IRT-based word difficulty; *p < 0.01. ^a^Intercepts of the words were allowed random effects. Effects were estimated with restricted maximum likelihood algorithms, under the assumptions of compound symmetry, first-order autoregressive, and unstructured covariance structures.

To correct estimates for within-subject temporal autocorrelation several covariance structures have been developed using *random-effects parameters* – additional unknown random variables assumed to affect the variability of the data. Jennrich and Schluchter (1986), proposed a general class of mixed linear models with structured and unstructured (*UN*) within-subject covariances, which can control for the possible effects of temporal autocorrelation. As there was no *a priori* hypothesis regarding the expected covariance structures among these temporal autocorrelations, the model was estimated under the assumption of an *UN* covariance matrix among the residuals, which constitutes the least restrictive set of assumptions that may be applied. Using *UN*, different parameters are estimated for the variance of each variable as well as different covariance parameters for each repeated measurement sequence (Jennrich and Schluchter, 1986). The nil χ^*2*^ and associated degrees of freedom were due to the extremely small magnitudes of these residual covariances (Residual*_UN_* = 8.117 × 10^-8^) and the “saturated” *UN* model of the residual covariance matrix, respectively. Thus, as compared with an equivalent General Linear Model (*GLM*) using Ordinary Least Squares (*OLS*), the parameter estimates for the effect sizes were within rounding error of each other.

In this MLM, the criterion was the absolute yearly frequency of word usage from 1850 to 2005 (operationalized as the percentage represented by each word of the total number of words then employed), and predictors were first year of word usage (*Word Debut*), the annual literacy rates of the UK population from 1850 to 2005, the natural logarithm (Ln) of time (to model the expected curvilinearity using the minimal model degrees of freedom), the item difficulties (δ) for each word, the *r*FPR for each word, the two-way interaction between population literacy and δ, the two-way interaction between *r*FPR and δ, the two-way interaction between δ and LN(time), the two-way interaction between *r*FPR and LN(time) and the three-way interaction between δ, *r*FPR and LN(time). Also estimated were the possible effects of any residual differences between the usages of words that were *not* attributable to δ by testing their main effects (operationalized as CLASS variables) and their temporal interactions at the end of each of the hierarchical MLMs constructed.

Due to small time intervals between measurements (1 year), parameter estimates for both time predictors and for the predictors involving the interaction of time with difficulty were very small. To compensate for this the estimates were recalculated by dividing all the years by 1000, changing the metric to occurrences per millennia. The advantage of this simple linear transformation (by which semipartial correlations and associated significance tests are not affected) is that the numerical magnitudes of the parameter estimates are visually increased, making the results more legible. All MLMs were conducted using PROC MIXED in SAS 9.3, and semipartial correlation coefficients were estimated using a beta version of UniMult 2 (for documentation on UniMult 1, see Gorsuch, 1991).

## Results

**FIGURE 1:**
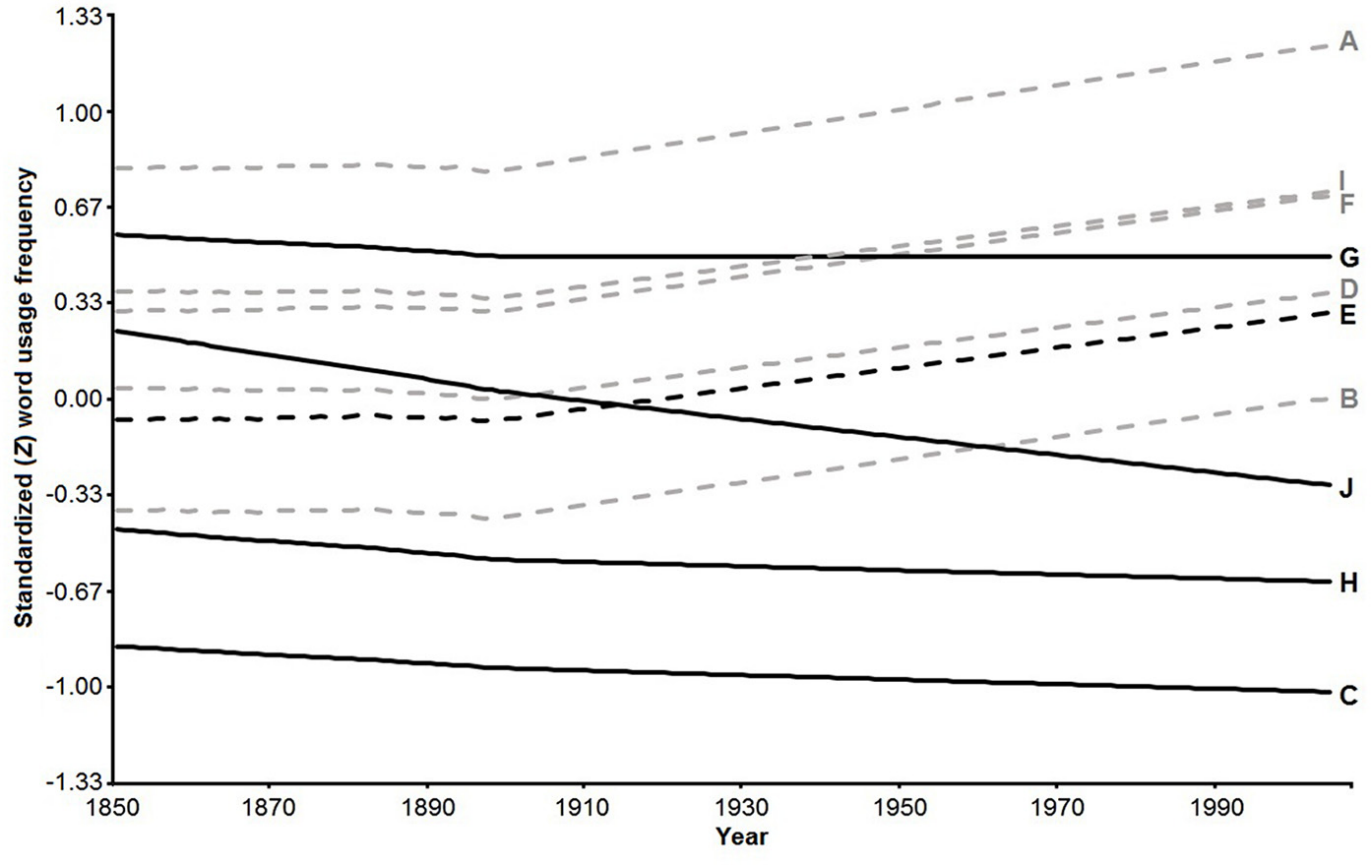
**Temporal trends of the 10 WORDSUM words based on the fitted mixed models**. *Solid lines* represent more difficult and *dashed lines* less difficult words. *Black lines* represent words that are more associated and *gray lines* less associated with rFPR (strength of association was based on a median split). WORDSUM codes are displayed to the right.

**Table 2** displays the semipartial correlations between word usage frequencies over time accounted for with each predictor, as assigned by hierarchical partitioning over variance (Type I Sums of Squares); all of these effect sizes were statistically significant at *p*< 0.001. As seen in **Figure 1**, words that are more difficult and for which the negative association between fertility and pass-rate is stronger present a more persistently negative trend over time. Easier words by contrast present a more persistently positive trend over time.

## Discussion

*More difficult* words presented *sharper* historical declines in usage over the 1850–2005 period, as indicated by the *negative* effect of the interaction between δ and LN(time). In line with this, the *positive* effect of the interaction between *r*FPR for each word and LN(time) indicates that words for which pass rates are *more negatively* associated with fertility are decreasing in usage over time, which is consistent with those being the *more difficult* words, as indicated by the MCV results presented above. Also consistent with predictions was the *positive* three-way interaction among each word’s δ, *r*FPR, and LN(time), which indicates that words that are both *more difficult* and for which pass rates are *more negatively* associated with fertility are *decreasing* over time. This suggests that δ had a *more negative* impact on usage of more difficult words over this historical period in cases where pass rates are *more negatively* associated with fertility. Therefore the *positive* two-way interaction between *r*FPR for each word and LN(time) applies primarily to the *more difficult* words. Generally speaking, words that are *more difficult* and for which pass rates are *more negatively* associated with fertility present a *sharper* historical decline in usage over the period under scrutiny; words that are *less difficult* and that exhibit a weaker association between fertility and pass rates present a *shallower* historical decline in usage over the same period. Also importantly, the *positive* main effect of time indicates a secular trend toward rising usage of all words over time, irrespective of population literacy. Additionally, the interactions discussed above clearly indicate that the less difficult words are increasing in usage over time, consistent with a Flynn effect, as can be seen in **Figure 1**.

The negative main effect of the δ parameter itself on word usage frequency simply indicates that more difficult words are generally used less frequently. Accordingly, the *small but positive* effect of *r*FPR on each word upon word usage indicates that words for which pass rates are *more negatively* associated with fertility are being used *less frequently* at the historical baseline than others. The *negative* effect of the residual interaction between δ and *r*FPR for each word upon word usage indicates counterintuitively that item difficulty had a *more negative* impact on overall word usage (i.e., independent of passing time) in words for which *r*FPR is weaker, unlike the time trends and main effects identified.

The *small but positive* main effect of literacy is the general baseline for the usage of all words in the sample, which *increased* as literacy increased, consistent with a Flynn effect. This effect is likely due to the general trend toward universal literacy (reaching an asymptote at 99% around 1900) amongst the reading public and concomitant vocabulary enrichment, especially with respect to less cognitively demanding words. The *negative* effect of the literacy × δ interaction indicates that more difficult words tended to *decrease* in usage with increasing literacy. This is likely also a consequence of the trend toward universal literacy, which presumably involved a higher proportion of persons of lower ability gaining access to literacy over time and demanding less difficult words in literature.

A large proportion of variance was attributable to the recency of each word’s introduction into the language, which was simply a control variable to statistically adjust for the effect of word age, but was not theoretically relevant to the hypotheses. The *negative* main effect of word debut year indicates that the *more recently* introduced the word, the *lower* the word usage, which is consistent with Curzan’s (2009) observations.

## Conclusion

These findings provide compelling evidence for the co-occurrence model, adding to the nomological net from which these and other predictions have been derived (Woodley et al., 2014a). They furthermore add weight to the argument that variance components should be taken seriously when considering the pattern of secular trends among indicators of IQ. The presence of opposingly directed trends on different WORDSUM items might even account for the results of studies finding no consistency in secular trends at the level of full-scale WORDSUM scores in GSS waves dating back to the 1970s (Beaujean and Sheng, 2010). These results replicate the findings of other studies on WORDSUM indicating performance declines on harder words, coupled with null-trends or improvements on less difficult ones, both at the level of the GSS survey waves (Bowles et al., 2005; Cor et al., 2012) and also among lexical databases (Haung and Hauser, 1998; Roivainen, 2014). The findings also build on this previous research by showing direct contributions to the decline in frequency among difficult words stemming from genetic selection.

It is predicted by the co-occurrence model that specific cognitive abilities should be increasing over the same period of time as *g* is declining. There are good theoretical reasons to expect that human social evolution should favor the role of cognitive specialization among individuals into different socioecological micro-niches, thus reducing intraspecific competition and enhancing the productivity of the social group by the economic mechanisms of Ricardo’s Law of Comparative Advantage (Woodley et al., 2013; Cabeza de Baca and Figueredo, 2014). Consistent with this is the finding of a direct contribution to the increase in frequency among less difficult words stemming from literacy, a theorized elicitor of the Flynn effect (i.e., Must et al., 2003; Marks, 2010). This is suggestive of increasing specialization with respect to the non-*g* ability variance associated with vocabulary.

It must finally be noted, however, that only the effect of genetic selection on word usage frequency over time was modeled here. Generational mutation accumulation is another factor likely influencing population *g* and potentially therefore word-usage frequency (Woodley of Menie, 2015). As mutation accumulation was not directly modeled, the effects of selection on word usage frequency likely underestimate the full effect of genetic changes taking place within Western populations over the time period covered in the present analysis.

## Conflict of Interest Statement

The authors declare that the research was conducted in the absence of any commercial or financial relationships that could be construed as a potential conflict of interest.
